# Radiographic and histologic effects of bone morphogenetic protein-2/hydroxyapatite within bioabsorbable magnesium screws in a rabbit model

**DOI:** 10.1186/s13018-019-1143-8

**Published:** 2019-04-29

**Authors:** Le Hoang Nam Dang, Yu Kyoung Kim, Seo Young Kim, Kuk Jin Lim, Ken Bode, Min Ho Lee, Kwang Bok Lee

**Affiliations:** 1 Department of Orthopedic Surgery, Chonbuk National University Medical School, Research Institute of Clinical Medicine of Chonbuk National University-Biomedical Research Institute of Chonbuk National University Hospital, Jeonju, South Korea; 2 Department of Dental Biomaterials and Institute of Biodegradable Materials, Institute of Oral Bioscience and BK 21 Plus project, School of Dentistry, Chonbuk National University, Jeonju, South Korea

**Keywords:** Magnesium screw, Bone morphogenetic protein 2, Hydrogen gas, Hydroxyapatite

## Abstract

**Background:**

Hydrogen gas formed by magnesium (Mg) screw corrosion can accumulate around the implant and create bone cysts, long-term osteolysis lesions, and bone healing delay. Thus, several authors currently do not recommend Mg implants for clinical use. In contrast, bone morphogenetic proteins (BMP)-2 have a very strong osteoinductive activity. The purpose of this study was to evaluate the effect of rhBMP-2/hydroxyapatite (HA) inside specially designed Mg cannulated screws in a rabbit femur model for hydrogen gas formation avoidance.

**Methods:**

Fifteen rabbits underwent randomly different cannulated Mg screw implantation in both distal femora; 30 femora were divided into three groups depending on the materials fill in the cannulated Mg screw: control group (Mg screw with no treatment), HA group (Mg screw with HA), and BMP-2/HA group (Mg screw with a composite BMP-2/HA). Plain radiography, micro-CT, and histological analysis were accomplished, and the ability to release BMP-2 of the screws was evaluated by immersion of both the screw with no treatment and screw with a composite BMP-2/HA into the SBF for up to 7 days.

**Results:**

X-ray assessment found the gas shadow around the implant was slightly smaller in the BMP-2/HA group than the HA and control groups at 8 weeks. Micro-CT analysis demonstrated statistically significant higher new bone formation in the BMP-2/HA group than the other groups, respectively, which also correlated with a decreased gas volume. Histological analysis showed higher osteointegration between implants and host femurs in the BMP-2/HA group than the HA and control groups at 12 weeks.

**Conclusions:**

This study indicates that the combination of BMP-2/HA within Mg screws enhances new bone formation and therefore has the potential to decrease the complications of hydrogen gas formation around these implants.

## Background

Biodegradable metals are promising options in solving the dilemma of stress shielding and secondary surgical interventions. Among these types of biodegradable metals, Mg has been widely and systematically investigated for biomedical applications. The rigidity of Mg is greater than ceramic biomaterials, and the elastic modulus and compressive yield strength of Mg are closer to those of natural bone than other generally used metallic implants [1]. Because of their similar elastic moduli, using Mg implants in boney applications would greatly reduce the implant’s stress shielding that could inhibit bone resorption. Furthermore, Mg ions are an essential element of the human organism, as the fourth most abundant cations in the human body, with approximately half of the total Mg stored in the bone tissue [2]. In addition, several authors report Mg to have good biocompatibility and may have stimulatory effects on the new bone formation. These properties assist the material’s consolidation into the surrounding bone and possibly allow full regeneration after degradation [1, 3, 4].

Unfortunately, the main disadvantage of Mg is a high corrosion rate with resultant hydrogen gas formation on contact with fluids [5]. This gas can induce complications such as superficial skin necrosis, and the gas bubbles can accumulate around the implant creating complications of bone cysts and long-term osteolysis lesions. These lesions can seriously delay the bone healing so that several authors are currently not recommending their clinical use [6–8]. In order to overcome this limitation of Mg implants, many techniques have been employed such as alloying, protective coatings, and surface treatments [9, 10]. The coating method was effective in diminishing the size of the voids in the surrounding bone but did not fully prevent degradation, although it was effective in avoiding the undesired initial burst release of gas [11]. Therefore, it is necessary to attempt different solutions to overcome these complications relative to hydrogen gas formation.

Bone morphogenetic proteins are members of the transforming growth factor-beta superfamily. Multiple bone morphogenetic proteins (BMP) are considered to have a crucial signaling role in chemotactic proliferation and differentiation of osteoprogenitor cells, thereby inducing bone formation [12]. Among the BMP, BMP-2 is the most extensively researched and has very strong osteoinductive activity [13]. Hydroxyapatite (HA) is used as a bone graft extender for posterolateral spinal fusion in humans [14]. It is also beneficial as a BMP-2 carrier because of its high attraction for BMP-2; BMP-2-adsorbed hydroxyapatite is a safe promoter of bone formation [15].

For the above reasons, this study used specially designed Mg cannulated screws with a BMP-2/HA delivery system inside the implants to evaluate the radiographic and histologic effects of this combination within a rabbit femoral model for avoiding hydrogen gas formation.

## Methods

### Experimental design

The selection of experimental animals, their management, and the surgical protocols were authorized by the Institutional Animal Care and Use Committee of the Chonbuk National University Laboratory Animal Center, Jeonju, South Korea (approved number: CBNU 2017-0013). Fifteen male New Zealand white rabbits (age, 2 months; body weight, 1.8–2 kg) were used, and the 30 distal femora were divided into three groups of 10 which had different Mg screw preparations randomly implanted into both distal femora: control group (Mg screw with no treatment), HA group (Mg screw with HA), and BMP-2/HA group (Mg screw with a composite BMP-2/HA). Four distal femora which contain samples from each group were collected at 8 weeks for micro-computed tomography (μCT) and histological assessment. The remaining nine rabbits (six samples from each group) were assessed with plain radiographs at 1, 2, 4, 8, 12 weeks and sacrificed at 12 weeks for μCT and histological assessment.

### Implant preparation

**Fig. 1:**
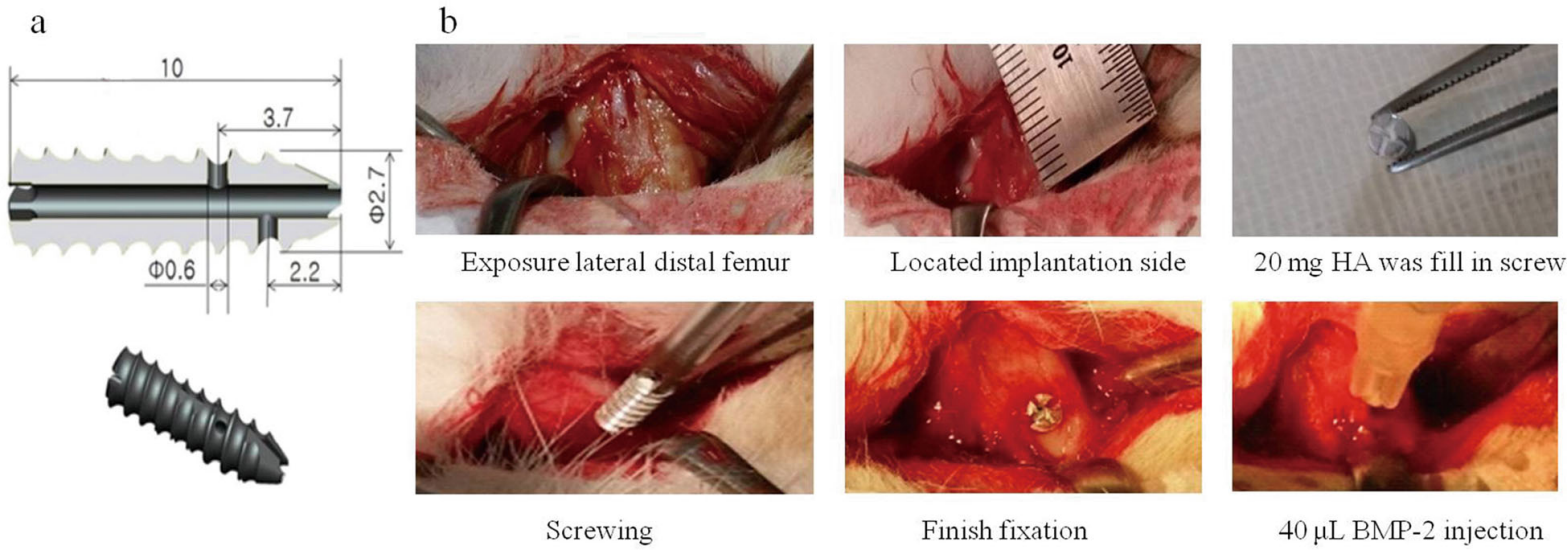
Experimental design. **a** Mg cannulated screw with special design. **b** Different portions of the surgical procedure

Mg alloy (Mg–Ca) cannulated screws were manufactured by U&I company, South Korea. These were headless screws: 2.7 × 10 mm, cannulated hole diameter 0.9 mm. Two 0.6 mm holes were placed in the body of the screw at a distance of 2.2 mm and 3.7 mm from the end of the screw, which connected the cannulated portion to the local environment and enabled elution of BMP from this section (Fig. 1a).

The BMP-2 used in this study was provided by Daewoong Pharmaceutical Co., Ltd. (Novosis®-dent, Gyeonggi, South Korea). Lyophilized BMP-2 was dissolved in 10 cm^3^ of distilled water to yield a concentration of 0.1 mg/ml. The doses of BMP-2 in this study were established based on the weight equivalent to the baseline dose that would be used in human undergoing spine fusion. The mean body mass, dose, and dose/weight assumed to humans were 70 kg, 1.5 mg, and 0.0214 mg/kg, respectively, resulting in values for the rabbits of around 2 kg, and the dose was 0.04 mg.

Hydroxyapatite, manufactured by Sigma-Aldrich company. (Sigma-Aldrich Korea 698-84 Maeng-ri, Wonsam-myun Cheoin-gu, Yongin City 17166 South Korea), was used as the carrier material, and 20-mg HA was placed into the screw canals in the HA and BMP-2/HA groups. The exact amount of HA was calculated by the difference in screw weight before and after fill-in of HA into the canal.

### Animal model

The animals were housed in separated cages under standard laboratory conditions and fed a standard diet. Rabbits were anesthetized with an intramuscular injection of a 3:1 solution of ketamine hydrochloride (Ketalar, Yuhan, Seoul, Korea) and xylazine (Rompun, Bayer Korea, Seoul, Korea). The lateral distal femurs were shaved and disinfected with iodine, and then infiltration anesthesia was induced by injection of 2% lidocaine (lidocaine-HCl, Hans, Seoul, Korea).

The lateral distal femoral metaphysis was surgically exposed by incisions through the skin and fascia lata. The periosteum was then elevated by performing a full-layer incision with an #11-blade, and using a periosteal elevator, the periosteum was gently dissected from the bone. The implant placement was 1 cm above the knee joint line on the lateral femoral condyle. The implants were manually screwed after drilling a hole of 2.5-mm diameter and 10-mm depth using a low-speed drill and saline irrigation. The HA and BMP-2/HA were then placed inside the cannulated screws for those groups. The soft tissue was repositioned and then layer closured using 4–0 synthetic absorbable multifilament suture materials (VicrylPlus Antibacterial, Ethicon, Somerville, NJ, USA) (Fig. 1b).

Postoperative antibiotics (Amikacin; 1.5 mg/kg body weight) were administered by daily intramuscular injection for 1 week. During the postoperative stage, the animals were allowed to move freely in their cages without external support. Skin sutures were removed at 10 days postoperative.

### X-ray radiographs and micro-computed tomography analysis

Anteroposterior and medial–lateral view X-rays of the rabbit’s treatment leg were collected to assess gas bubble (radiolucent/dark color) and bone formation (radiopacity/gray color).

The bone specimens including screws were harvested by transaxial cutting 0.5 cm above and below the screw position. Block biopsy specimens were quantitatively analyzed using micro-CT (Skyscan 1076) at 100 kV and 100 μA, respectively, with 240 ms of exposure time. The specimens were immersed in 10% neutral buffer formalin solution during the time of imaging. The bone and gas formation volumes were estimated by the phantom in Hounsfield Units (HU) using CTAn software (Skyscan). The image from micro-CT scan was reconstructed by DataViewer software and reconstructed images were continued to be analyzed with CTAn software; the area to be analyzed from the reconstructed images for trabecular is specified by the region of interest (ROI), which is typically selected adjacent to the around screw and endocortical surface for trabecular bone. ROI selection is achieved using a freehand drawing tool, and auto-interpolation between the different ROI levels produces the total volume of interest (VOI) for all frames selected.

Gas volume was quantified on the same procedure with bone volume, but different in bone mineral density calibration value.

### Histological analysis

After completing micro-CT scanning, the fixed distal femur blocks were immersed in Villanueva solution (Polysciences) for 3 days and dehydrated in a graded series of ethanols and acetone 100%, and then embedded in methyl methacrylate resin. Transaxial sections (approx. 0.5 mm thickness) were cut, ground to 70 μm thickness, and polished for hard tissue evaluation by optical microscopy (EZ4D, Leica).

### The ability to release BMP-2 of the screw analysis

To evaluate the ability to release BMP-2 of the screws, the screw with no treatment and screw within 40 μg BMP-2/20 mg HA were incubated in 1.5 ml micro-tube containing 1 ml of simulated body fluid (SBF, pH 7.4), under 100 rpm at 37 °C. At predetermined time intervals of 1, 2, 3, 6, 12 h and 1, 2, 4, 7 days, the supernatant was completely removed and replaced with a fresh SBF solution. Samples were stored at − 20 °C until the test. The amount of released protein was measured using the Human BMP-2 enzyme-linked immunosorbent assay (ELISA) kit. The absorbance of specimens was measured at a wavelength of 450 nm with ELISA reader (Model Spectra MAX PLUS, Molecular Devices, Sunnyvale, CA). The experiments were run in triplicate per time point.

### Statistical analysis

The mean values and standard deviations for new bone formation and gas formation around screws data were calculated for each group. Shapiro–Wilk test was performed to test data for normal distribution. An independent two-sample *t* test (when the data had normal distribution) and Mann–Whitney *U* test (when data had non-normal distribution) were conducted to compare differences between the groups. All tests were performed using SPSS software, version 20.0 (SPSS, Chicago, IL, USA), and *p* values under 0.05 were considered statistically significant.

## Results

All 15 rabbits survived the surgical procedure, and there were no wound infections, local inflammation, or morbidity in any of the animals.

### Radiographic evaluation

**Fig. 2:**
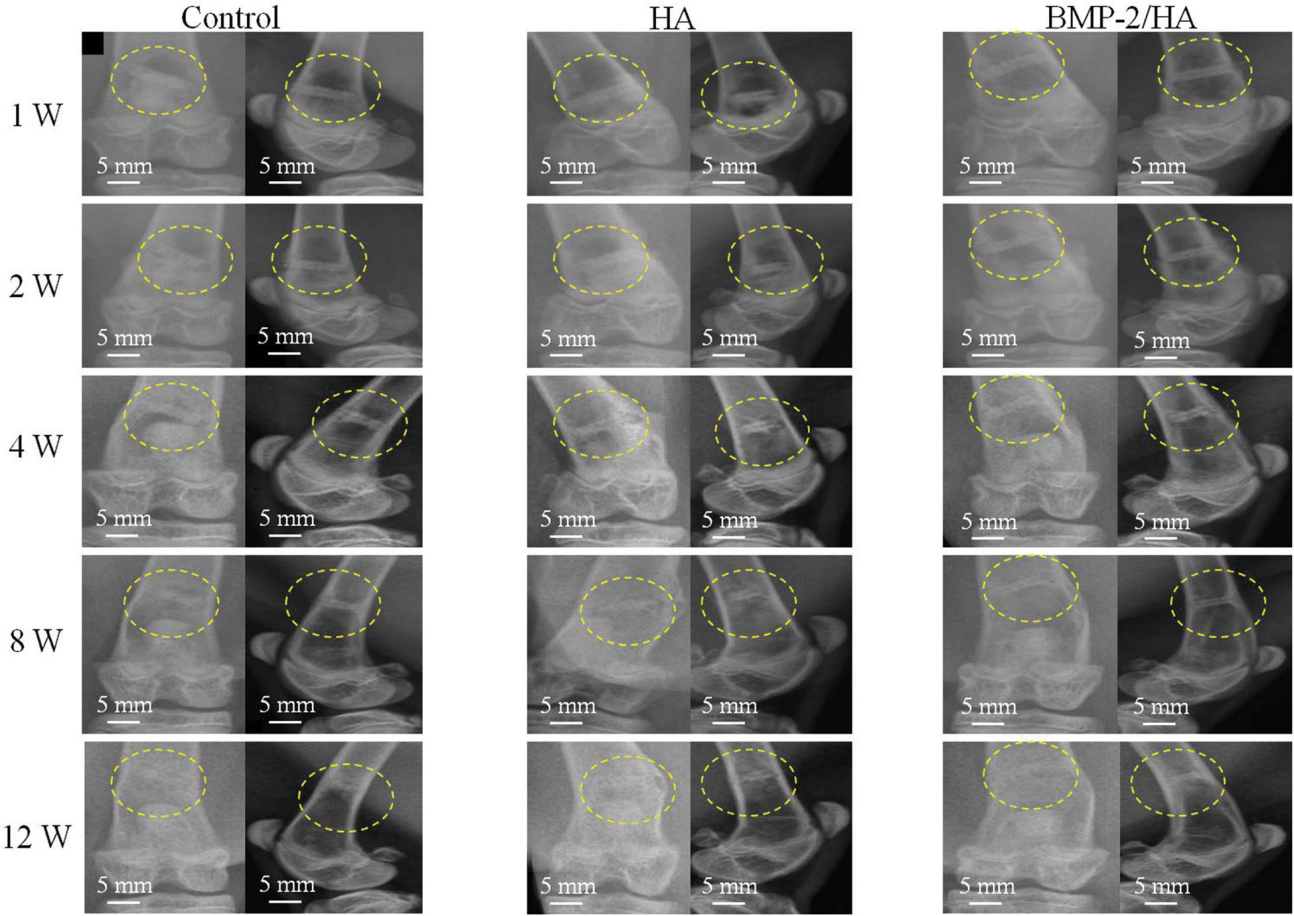
Anteroposterior and lateral view radiographic images of the experimental region. Images were taken from 1 to 12 weeks postoperative. The yellow circles indicate the position of the screw

Figure 2 comprises radiographs showing the postoperative change in each group; the yellow circles indicate the position of the screw. No evidence of implant migration was observed on radiographic evaluation up to 12 weeks.

In all animals, early gas formation around the screws was evident by 1 week postoperative (radiolucent areas around screw), and the gas expansion areas were larger after 2 weeks. At the 4-week time point, newly formed bone appeared (radiopacity areas), which sequentially replaced the gas around the implant. Of note, at 8 weeks, the radiopacity areas around the implant were slightly more in the BMP-2/HA group than the HA and control groups. This difference was especially evident by 12 weeks, as the control and HA groups had continued gas presence while the BMP-2/HA group had an apparent complete replacement of gas with newly formed bone. Likewise, the radiographic density appeared to be greater in the control/HA groups than in the BMP-2/HA group, respectively.

### Micro-CT evaluation

**Fig. 3:**
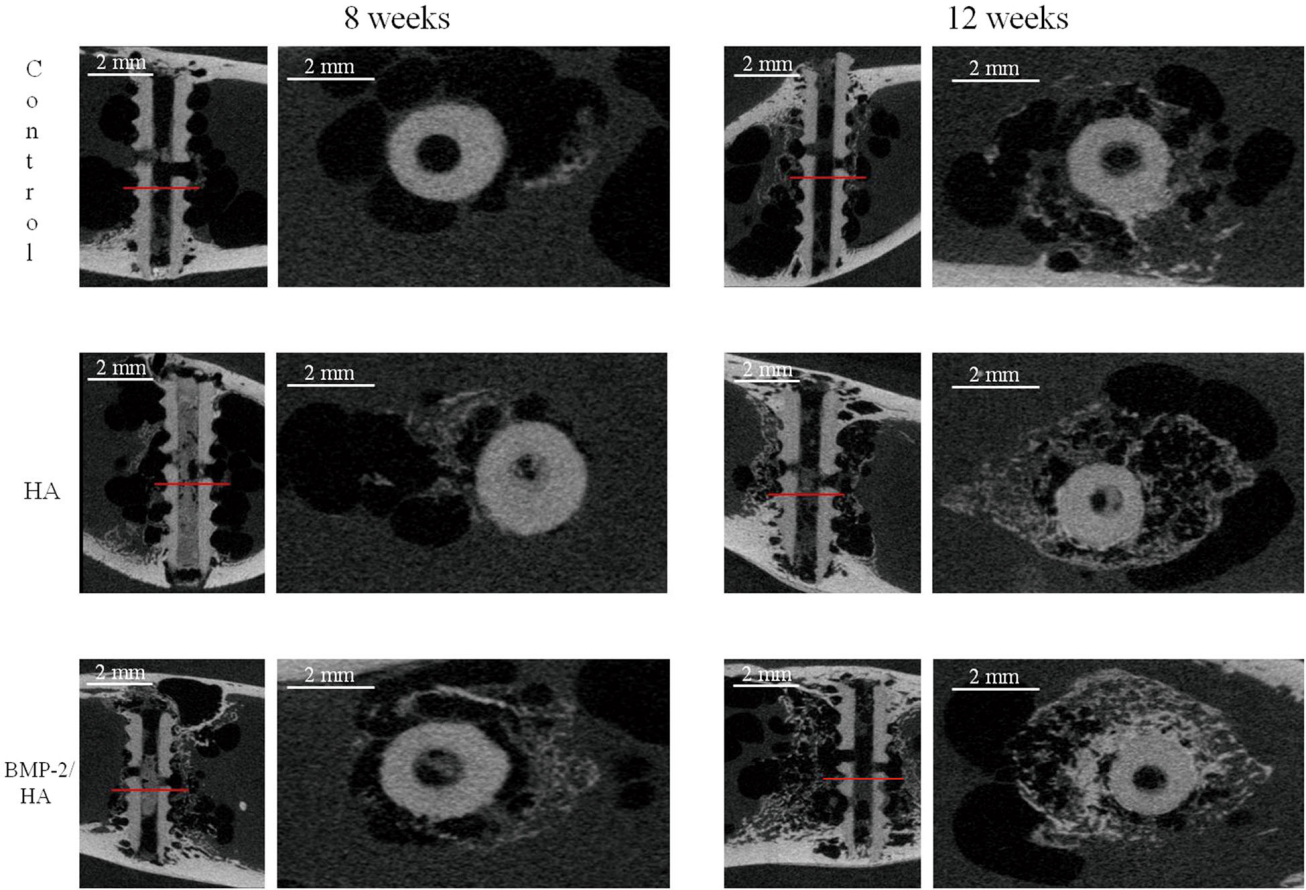
Coronal and sagittal plane micro-computed tomography images at 8 and 12 weeks postoperative. Red lines indicate the position of the sagittal plane from the coronal plane of the screw

In accordance with the radiographic findings, the coronal and sagittal plane micro-computed tomography images with red lines which indicate the position of the sagittal plane from the coronal plane of the screw, at 8 weeks and 12 weeks (Fig. 3), demonstrated the quality and quantity of new bone formation around the implant. The BMP-2/HA group showed a higher density of bone than the other two groups with bone formation more approximated to the implant surface in the BMP-2/HA group, respectively.

**Fig. 4:**
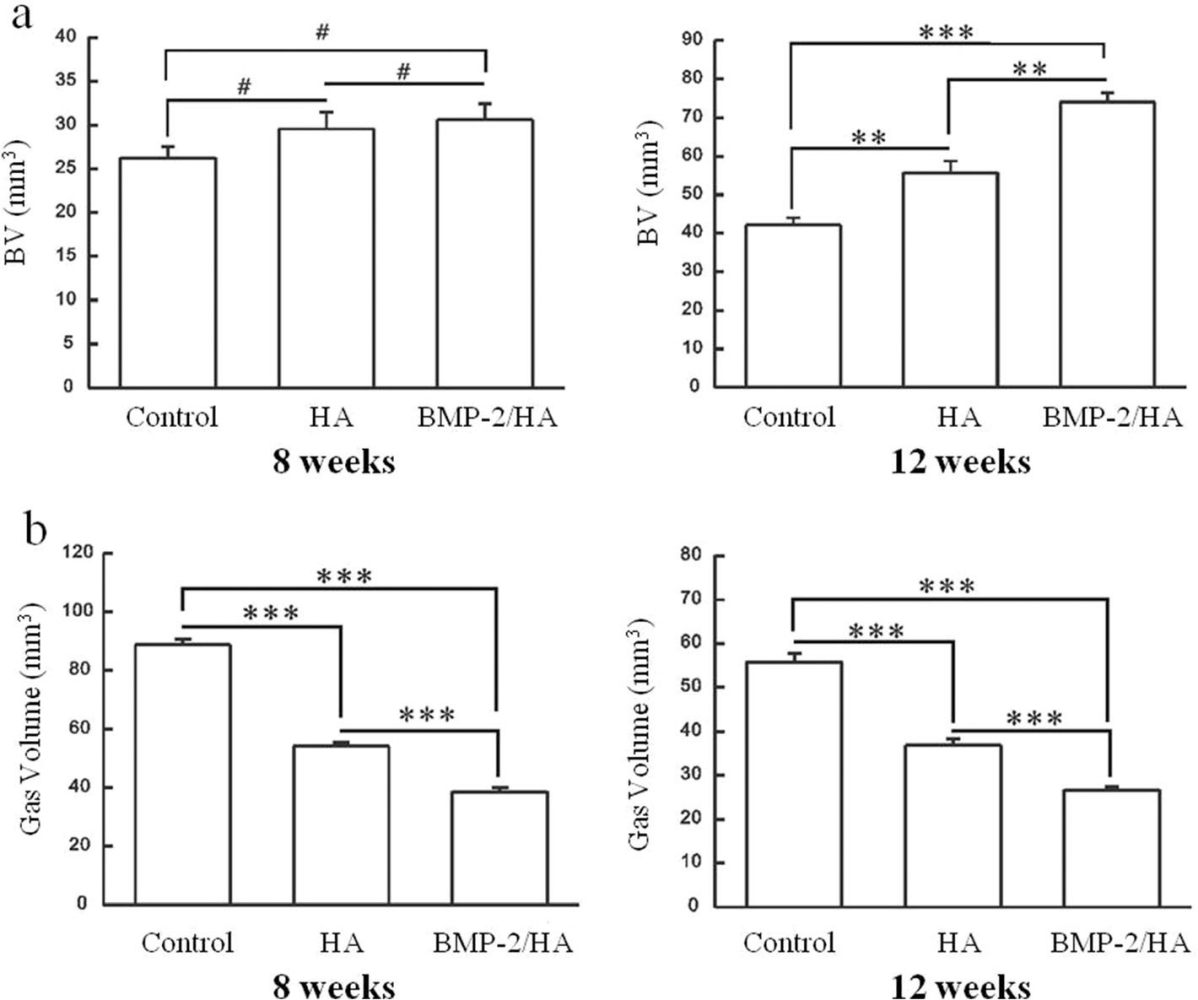
Quantitative analysis of micro-computed tomography. **a** Parameters of the new bone volume formation around implants in the control, HA, and BMP-2/HA groups at 8 and 12 weeks. **b** Parameters of the gas volume formation around implants in the control, HA, and BMP-2/HA groups at 8 and 12 weeks. Two asterisks indicate significant differences with *p* < 0.01, three asterisks indicate significant differences with *p* < 0.001, and number sign denotes no significant differences with *p* > 0.05

At 8 weeks, similar new bone formation results were seen between the three groups, with higher bone volumes in the BMP-2/HA group than in the control/HA groups, but this difference was not statistically significant (Fig. 4a).

At 12 weeks, the micro-CT analysis demonstrated a significantly higher new bone volume formation in the BMP-2/HA and HA groups when compared to the control group (*p* < 0.001 and *p* < 0.01, respectively). The newly formed bone volume was also significantly higher in the BMP-2/HA group compared with the HA group (*p* < 0.01) (Fig. 4a).

Furthermore, according to the micro-CT analysis at 8 and 12 weeks, the volume of gas formed was significantly lower in the BMP-2/HA group compared with the other groups (*p* < 0.001), respectively. Also, the volume of gas formed around implants decreased from 8 to 12 weeks in each group (Fig. 4b).

### Histological evaluation

**Fig. 5:**
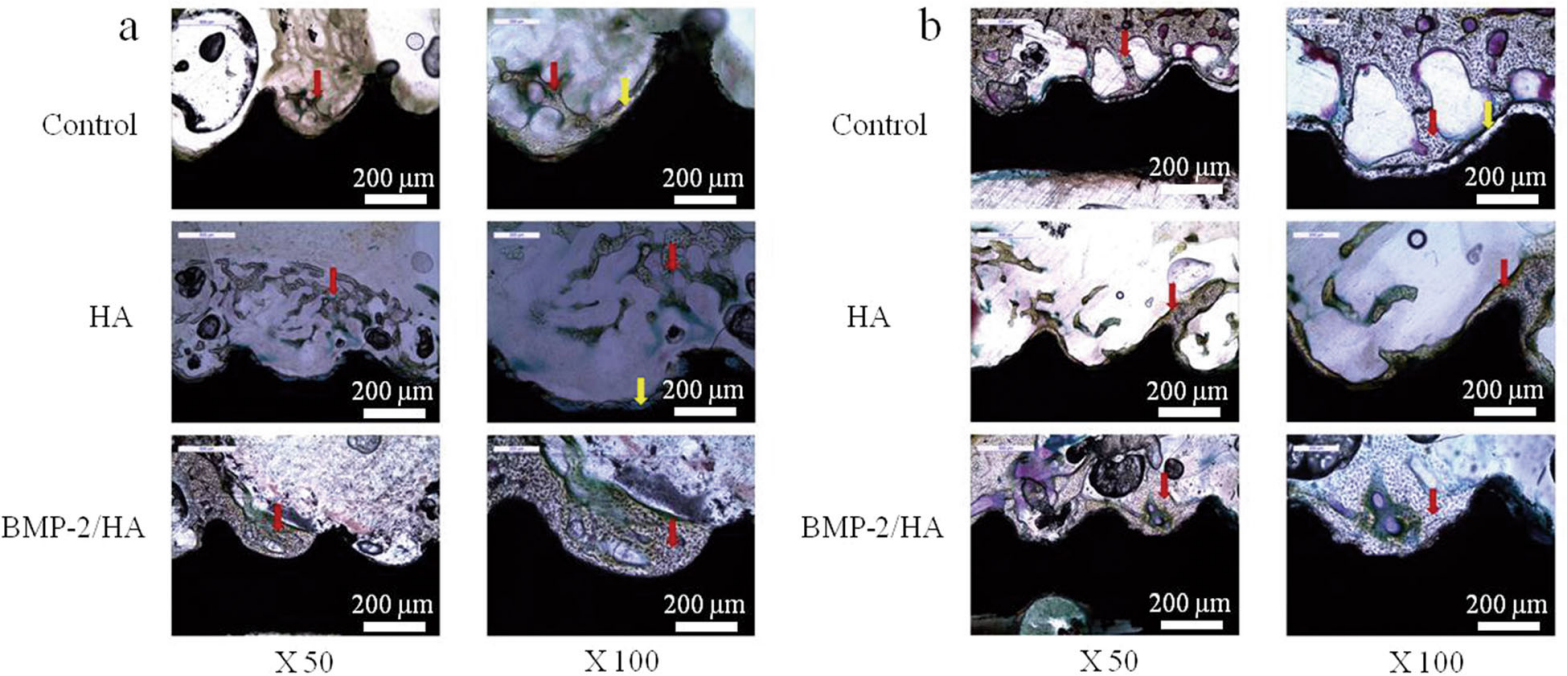
Histological images postoperative (Villanueva bone stain). **a** At 8 weeks. **b** At 12 weeks. Red arrows indicate newly formed osteocyte and yellow arrows indicate the gap between new bone layers and implant surface

At 8 weeks postoperative, histological analysis showed an implant and new bone interface in all groups; red arrows indicate newly formed osteocyte. However, the integration between implant and new bone within the BMP-2/HA group was stronger than the HA and control groups, as the newly formed bone in the HA and control groups was scattered along the screw surface (Fig. 5a).

At 12 weeks postoperative, increased new bone formation was observed around implants in all groups. However, at the 8- and 12-week time points, the BMP-2/HA group showed stronger integration between implant and new bone when compared with the other groups. With yellow arrows indicating the gap between new bone layers and implant surface, small gaps were observed between the implant surface and newly formed bone in the HA group which was larger in the control group (Fig. 5b).

### The ability to release BMP-2 of the screw evaluation

**Fig. 6:**
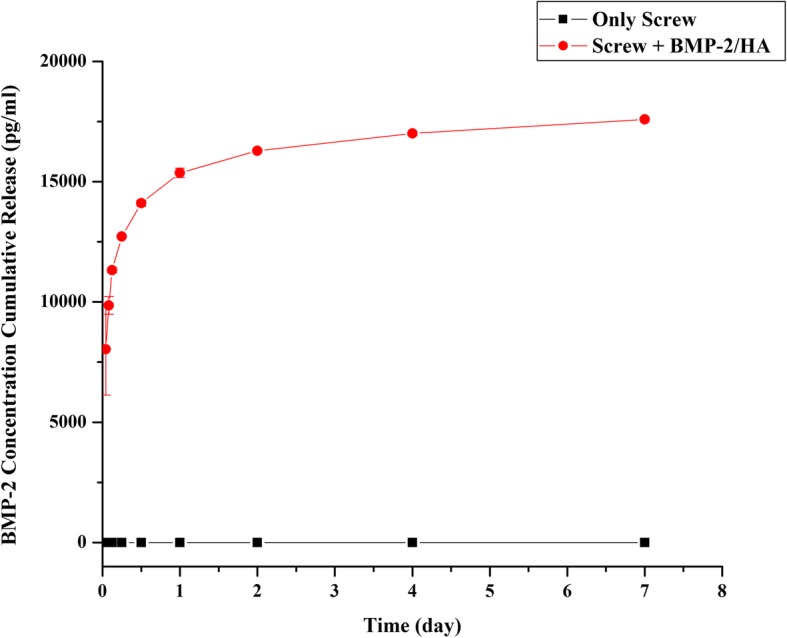
BMP-2 released profile of the screw treatment with a composite BMP-2/HA compared with the screw with no treatment

Figure 6 shows results demonstrating the measured quantities of BMP-2 released with time between the screw with no treatment and screw with treatment within 40 μg BMP-2/20 mg HA. During 7 days, the concentration of released BMP-2 in the screw treatment within BMP/HA was above 8032.593 ± 1900.805 pg/ml, and the results revealed a rapid increase in BMP release during the first 1 h and release profile continued to increase over the 7-day period; the total amount of BMP-2 released after 7 days was 17,594.97 ± 90.3132 pg/ml, while in the screw with no treatment was 0 pg/ml at each tested time point.

## Discussion

The complications of hydrogen gas formed from Mg alloy screws remain controversial. Most research has been within preclinical studies which report gas formation during degradation without specific complications [8, 16]. In contrast, according to Noviana et al., the authors showed the adverse effect of excessive hydrogen gas evolution to the survival rate of rats implanted with magnesium implants; the persistent presence of gas cavity causes prolonged discomfort and disturbs the balance of blood cell parameters which in turn decreases the survival rate [17]. On bone healing, only a few preclinical and clinical studies have shown the side effects of hydrogen gas production. A hypothesized explanation is that the space-consuming gas pockets raise the inner mechanical pressure, which then inhibits the initial cortical bone healing process. These gas bubbles also potentially decrease osteocyte integration with the implant surface. Therefore, some authors do not recommend Mg alloy screws for clinical use [6, 7, 18]. We hypothesized that a Mg cannulated screw delivery system with BMP-2/HA would decrease the limitations of Mg screws relative to hydrogen gas formation.

According to radiographic evaluation, gas formation was first observed at 1 week postoperatively and continued to increase until 8 weeks; after that, the radiolucent area decreased. This result could be explained as gradual new bone formation which replaced the gas space around screws. Another possible explanation is the gas bubble was absorbed into the surrounding tissues [19]. Several studies describe this gas formation through implant degradation which develops in the first week and then decreased over time [8, 11]. In an in vivo study, Li et al. observed gas shadows in the soft tissue and bone marrow cavity around Mg alloy implants during the early implantation period which disappeared 2 months after implantation [20]. However, in our study, the micro-CT scans revealed the gas bubble still remained until 12 weeks (Fig. 3). This could be explained through the implant’s local environment and available blood flow, which differentially clear the gas from the implant site. In the present study, the implants were placed in a bone marrow environment, which has a lower water content and blood flow than muscle or subcutaneous tissues thus resulting in potentially slower absorption.

The micro-CT images, at 8 and 12 weeks postoperative, found new bone formation around the implants which replaced the gas bubble space. However, the properties of that circle of bone formed were different between the three groups. Within the control group, the newly formed bone’s density appeared to be higher in the peripheral versus the central portion, but in contrast, the other two groups showed opposite results (Fig. 3). This likely relates to the material within the canal of the screw (HA or BMP-2/HA) which is released to the environment around the screw through the cannulated holes and the holes that were placed into the body of the screw. Furthermore, the new bone volume at 12 weeks was significantly higher in the BMP-2/HA group than that in the HA and control groups. At 8 weeks, the bone volume was also higher in the BMP-2/HA group than the other groups but not at a statistically significant level at that time (Fig. 4a). In addition, the volume of gas formed around the screw according to micro-CT analysis showed a significantly less amount from the BMP-2/HA group to the HA and control groups at both 8 and 12 weeks postoperatively (Fig. 4b). This shows the potential correlation between the increase in new bone formation and the associated decrease in gas volume.

In addition, at 8 weeks, histological images revealed an improved integration between the newly formed bone and the implant surface within the BMP-2/HA group. The HA group showed a gap between the bone–implant surface while the control group’s bone–implant gap was larger and new bone formation was scattered (Fig. 5a). At 12 weeks, new bone formation improved in the three groups, but the control group’s gap remained. A gap was not observed in the HA group; however, the bone/implant interface was thinner and more scattered when compared with the BMP-2/HA group (Fig. 5b).

The effect of BMP-2 on bone regeneration has been established in both preclinical studies and clinical trials [21]. According to this study’s promising results from radiographs, micro-CT, and histological analysis, the BMP-2/HA delivery system shows remarkable efficiency in forming new bone around the implant when compared with other groups. BMP-2 has proved to be one possible strategy for inducing bone formation, but the successful application of BMP-2 depends on the optimal therapeutic dosage, delivery system, and local circumstances for bone repair; these factors are still under examination [21]. The BMP-2 dose must be sufficient for adequate bone formation, yet not cause the known complications of ectopic ossification, inflammatory reaction, and pain [22]. This inflammation with abnormal bone formation has been observed in rats after 2 weeks in critical-sized femoral bone defects treated with high doses of BMP-2 (> 150 μg/ml) [23]. Therefore, in our study, we used an average 40 μg/mL dose of BMP-2 based on our previous research [24, 25] and the baseline dose used in human spinal fusion. No side effects such as skin infection, necrosis, and local inflammation were observed for up to 10 days, which means that acute complications relative to wound site do not occur; besides that, we checked the surgical position on the animal each time we fed it during the period of postoperative to sacrificed animals to harvest the samples as well as during the sample harvesting operation and we did not observe any evidences of local infections or inflammations as well as ectopic bone.

The carrier also plays an important role in the effectiveness of BMP-2. The carriers should be easily sterilized, should be biodegradable, and should have no immunogenicity. They should also provide an osteoconductive matrix that stabilizes the release of BMPs and thereby lowers the required dose [26]. We choose HA as the BMP-2 carrier since it is capable of directly bonding to bone and can improve corrosion resistance as well as restrain the pH increase. BMP-2 adsorption onto HA can also enhance interfacial strength and improve contact between the HA and surrounding bone. This promotes greater bone regeneration around the screw than HA alone [27]. This study had similar new bone volume formation as other investigations using the same HA carrier for BMP-2 [15, 28, 29].

Screws used in our study are cannulated screws; interestingly, cannulated screws are commonly performed as minimally invasive techniques for the treatment of articular and periarticular fractures. Especially when formal open reduction and internal fixation is contraindicated because of associated soft-tissue compromise and swelling, they can be inserted by way of limited open or percutaneous techniques in many cases to protect the skin and healing. Accurate fracture reduction can be attained through insertion of cannulated screws over a guide pin, resulting in provisional stability of the fracture and reduction of the chance of angulation error [30].

Cannulated headless screws made of magnesium alloy have been available on the market for osteosynthesis; since 2015 in Korea, the Mg–5Ca–1Zn alloy screws are commercialized by U&I Corp. (Gyeonggi-do, Korea), and they are now available in the market as K-MET bioresorbable bone screws, and in Germany, since 2017, the magnesium cannulated screws (alloy MgYREZr) are now commercialized by Syntellix (Hanover, Germany) under the name Magnezix [31]. Plaass et al. reported a clinical study, wherein the Mg–Y–RE–Zr screw was compared with the standard titanium screw for fixation of a modified distal metatarsal osteotomy in 26 patients with a symptomatic hallux valgus [32]. Lee et al. reported the results of a long-term clinical study of Mg–5Ca–1Zn alloy screws in 53 distal radius fracture fixation cases [19]. Moreover, some clinical case reports were published to introduce the application of magnesium cannulated screw in clinical. Both authors, Baver et al. and Biber et al., performed magnesium cannulated screws to lateral malleolar fixation. Similarly, Meier et al. used magnesium cannulated screws for scaphoid fracture and Leonhardt et al. used it for fixation of fractures of the condylar head of the mandible [7, 33–35]. Despite magnesium cannulated screws have been available on the orthopedic field, complications relative to gas formation around implant have been reported. On the clinical study of Meier et al., they reported about extensive bone cyst and the long time period for bone healing when they use magnesium cannulated screws for scaphoid fracture and they concluded that cannulated magnesium screw is currently not recommended for clinical use in scaphoid fracture [7].

Moreover, the current literature reveals that a wide range of coating materials and coating formation techniques are employed to enhance the corrosion resistance of Mg-based alloys. However, most of the in vivo or in vitro studies in the literature investigated the corrosion behavior of the coated samples over a period of maximum of a few days or weeks. The results from the studies may not be used to determine the long-term corrosion performance of the same sample; as the coatings will disappear with time, the corrosion process will accelerate and complications related to gas formation around implant will occur [36]. Even with good coating methods, it cannot completely reduce corrosion rate, which means gas formation around screw still was observed; thus, most coatings for Mg alloys described in the literature have not been developed for medical applications, and no commercial products are available in the biomedical devices sector [37].

According to the ability to release BMP-2 of the screw evaluation, after screw within composite BMP-2/HA immersed in the SBF, the BMP-2 concentration was found and continued to increase over time (Fig. 6). These results verify that the BMP-2 within the canal of the screw is released to the environment around the screw through the cannulated holes and the holes that were placed into the body of the screw. Therefore, besides the advantages of conventional cannulated screw that we mentioned above, the screws used in our study have special design; two holes were placed in the body of the screw, which connected the cannulated portion to the local environment and enabled slow elution of materials from this section in parallel with the formation of gas around the screw processing; and the material in this case is BMP-2, which was demonstrated with powerful osteoinductive and inducing bone formation ability. Thus, the combination of special design in our cannulated screw and BMP-2 is possible to overcome the complications related to gas formation around magnesium screws.

There are several limitations to our study. First, this study lacked in vitro investigation to examine the interaction between BMP-2 and Mg to determine the effect of BMP-2 on Mg corrosion behavior and biomechanical properties. Instead, this study placed a priority on imaging and histology to observe and quantify the newly formed bone around Mg implants. Another limitation relates to the singular BMP-2 dose used in this study, which did not compare the effect of different BMP-2 doses on bone formation such as fracture healing, bone defect, or distraction osteogenesis [28, 38, 39]. Future work is required to determine the most ideal BMP-2 dose and other carriers, such as absorbable collagen, β-tricalcium phosphate, and gelatin.

## Conclusions

The placement of BMP-2/HA within a cannulated Mg screw enhanced the bone formation ability which replaced the gas void around the implants. Thereby, this combination has the potential to limit the complications of hydrogen gas accumulation. Further research is required to analyze BMP-2 doses and the in vitro interactions between BMP-2/HA and Mg to include biomechanical evaluations compared to other systems.

## Abbreviations

BMP: Bone morphogenetic protein
HA: Hydroxyapatite
Mg: Magnesium
ROI: Region of interest
VOI: Volume of interest
μCT: Micro-computed tomography
